# Root canal disinfection of immature dog teeth with apical periodontitis: Comparison of three different protocols

**DOI:** 10.4317/jced.51475

**Published:** 2014-10-01

**Authors:** Soledad Rodríguez-Benítez, Carlos Stambolsky Guelfand, Milagros Martín-Jiménez, Juan-José Segura-Egea

**Affiliations:** 1 Department of Stomatology (Endodontic Section), School of Dentistry, University of Sevilla, C/ Avicena s/n, 41009-Sevilla, Spain

## Abstract

Objectives: The present in vivo study was designed to assess the efficacy of 3 root canal disinfection protocols in immature dog teeth with apical periodontitis (AP). Material and Methods: Forty immature premolars with pulp necrosis and AP of five Beagle dogs were used. Three experimental disinfection protocols were established. After irrigation with 40 ml 5.25% sodium hypochlorite using the Endovac system, in Group 1 canals were flushed with QMix solution; in Group 2, canals were flushed with QMix solution and 2% chlorhexidine gel dressing was placed for two weeks; and in Group 3, triantibiotic paste dressing was placed for two weeks. Canals were sampled after periapical lesions were radiographically visible (S1), after the first disinfection session (S2) and, in groups 2 and 3, after dressing (S3). Results: After the first session of the disinfection protocol (S2), there was significant (p < 0.05) bacterial reduction in the three experimental groups. Microorganisms were absent in 100% of S2 samples in groups 1 and 2, and in 75% of group 3 (p > 0.05). After dressing, 87.5% of the S3 samples showed increased bacterial count: in group 2, CFU counts (median = 891) were significantly higher than in group 3 (median = 18) (p = 0.03). Conclusions: In immature dog teeth with AP, root canal irrigation using QMix solution, with or without chlorhexidine gel dressing, or a triantibiotic paste dressing, provides the same level of disinfection than irrigation with 5.25% sodium hypochlorite alone in only one session.

** Key words:**Apical periodontitis, chlorhexidine, Endovac, immature teeth, QMix solution, root canal disinfection, triantibiotic paste.

## Introduction

The main etiologic factor of pulp and periradicular pathosis are microorganisms invading into dentin and further into the root canal system of the teeth (1). Therefore, the goal of endodontic therapy is to eliminate the bacteria and their products and byproducts from the root canal and the infected dentin. This is particularly important in teeth with pulp necrosis and periapical injury (2).

Because of the anatomic complexity of root canal systems, organic waste, pulp tissue remnants, inorganic debris and bacteria remain into the root canals after mechanical preparation alone (3). For that reason, irrigation solutions are essential for successful debridement, cleaning and disinfection of the root canals after mechanical shaping procedures. However, there are cases in which bacteria are left in the canal system even after a thoroughly instrumentation and irrigation of the root canals (2). This risk is higher when the biomechanical preparation is more difficult, such as in immature teeth with apical periodontitis with thin divergent or parallel dentinal walls (4).

Taking into account the difficulty of root canal system disinfection in immature teeth and considering the polymicrobial nature of endodontic infection, it has been proposed the use of a mixture of antibiotics dressing [ciprofloxacin, metronidazole and minocycline] to reduce the diverse root canal system microflora (5). But considering that this intracanal dressing can produce crown discoloration (6), an alternative protocol for immature teeth with apical periodontitis using apical negative pressure irrigation [EndoVac; Discus Dental, Culver City, CA] has been suggested (7). This new protocol has been shown to provide similar bacterial reduction that apical positive pressure irrigation plus triantibiotic intracanal dressing in immature dog teeth with apical periodontitis (7).

Recently, a new root canal irrigant [QMix, Dentsply Tulsa Dental, Tulsa, OK] containing a mixture of the bisbi-guanide antimicrobial agent chorhexidine, the calcium-chelating agent EDTA, and a surfactant detergent, have been found to be effective for smear layer removal and against bacterial biofilms (8). QMix solution should be considered as a potential new approach for immature teeth with apical periodontitis.

The present in vivo study was designed to compare the efficacy of 3 different protocols for root canal disinfection in immature dog teeth with apical periodontitis: 5.25% sodium hypochlorite and apical negative pressure irrigation combined with QMix alone, QMix plus chorhexidine gel or triantibiotic paste. The null hypothesis tested was that there are no differences in the ability of these three protocols to reduce the root canal microflora.

## Material and Methods

The study protocol was reviewed and approved by the Ethical Committee of the University of Sevilla [Spain].

Forty double-rooted second and third maxillary and mandibular immature premolars of five Beagle dogs, approximately 5 months old and weighing approximately 11 kg each, were selected for this study. The teeth were randomly divided into 3 experimental groups, 10 teeth each one, and two control groups, 5 positive controls [infected but untreated] and 5 negative controls.

**Figure 1:**
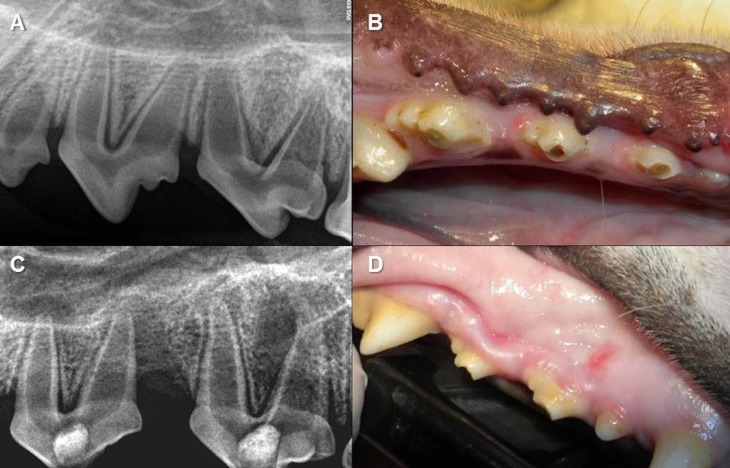
A) Initial periapical radiograph to confirm incomplete root formation and open apices; B) Root canals were left exposed to the oral cavity to allow microbial contamination; C) Periapical radiograph confirming apical periodontitis; D) Sinus fistula in relation with a tooth with chronic apical periodontitis.

Before the start of the study, all teeth were examined radiographically [Kodak RVG 6100® Digital Radiography System, Carestream Health, Inc. Rochester, NY, USA] using radiograph paralelling devices [Dentsply Rinn, Elgin, IL, USA] to confirm incomplete root formation and open apices (Fig. 1).

The dogs were preanesthetised with an endovenous injection of tiletamine hydrochloride: zolazepam hydrochloride [Zoletil 100, 0.1 mL/kg body wt.; Virbac España, S.A., Spain] and were kept with inhalation anesthesia with Isoflurane [Isoflo, Abbott Laboratories Ltd., Berkshire, UK.]. Local anesthetic Lidocain [Lidocaína hiperbárica Braun 5%, B. Braun Medical S.A, Barcelona, Spain] was also used during all procedures to prevent the animals from suffering.

The aim of the first treatment session was to infect the pulps of randomly selected immature dog teeth. Coronal accesses were done with a round diamond sterilized bur #12, at high speed with copious water cooling under nonaseptic conditions. Then, the pulp was disrupted with a #20 sterile stainless steel Hëdstrom file [Colorinox, Dentsply Maillefer, Ballaigues, Switzerland] and the root canals were left exposed to the oral cavity for 7 days to allow microbial contamination. This procedure was repeated for each individual dog (Fig. 1). The coronal accesses were sealed with Cavit [ESPE, Norristown, PA, USA] with no intracanal dressing to induce apical perio-dontitis, which occurred within 15 to 25 days.

**Figure 2:**
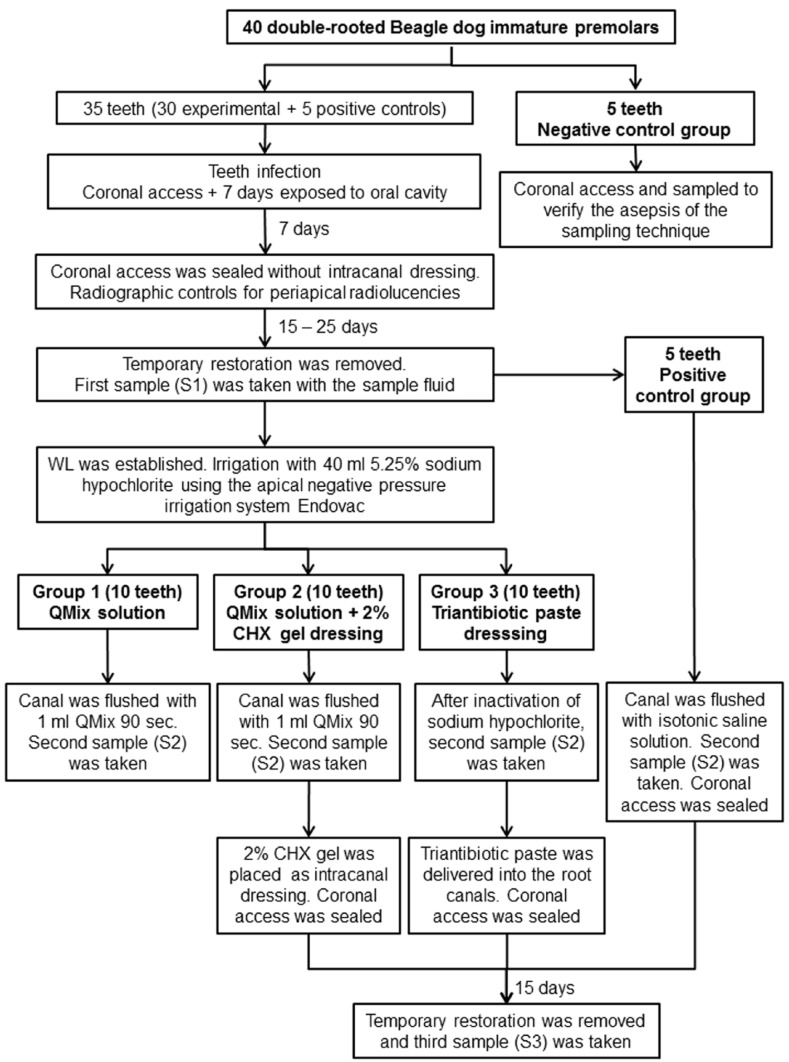
Flow chart showing the different steps of the experimental protocols. CHX: chlorhexidine. WL: working length.

At the end of this period, radiographs were taken to confirm periapical radiolucencies (Fig. 1). Once the lesions were radiographically visible, 30 teeth were randomly assigned to 3 experimental groups, 10 teeth each one, according to the intracanal disinfection protocol (Fig. 2). Ten teeth were assigned to the control groups [5 negative and 5 positive].

The intracanal disinfection protocol carried out in each of the experimental groups was as follows: all teeth were firstly disinfected with 40 ml 5.25% sodium hypochlorite [NaOCI] using the apical negative pressure irrigation system Endovac [Endovac; Discus Dental, Culver City, CA, USA] (7), then Group 1 received a final flush of QMix; Group 2 received a flush of Qmix, followed by intracanal dressing with 2% chorhexidine gel; Group 3 received an intracanal dressing with a triantibiotic paste consisting of metronidazole, ciprofloxacin, and minocy-cline. The three experimental groups were tested in each animal, and the experimental protocols were performed in alternate quadrants in a randomized manner.

All teeth were isolated with a rubber dam, and the operative field was disinfected with 30% hydrogen peroxide until no bubbling of the peroxide occurred. All surfaces were then coated with tincture of iodine and allowed to dry. The temporary restoration was removed with a round sterilized bur #12 in a high-speed handpiece. Sterile saline was then used to flush any debris from the pulp chamber. Sterile cotton pellets were then used to dry the pulp chamber before the placement of 1 ml of the sample fluid Schaedler Anaerobe Broth with hemin and vitamin K1 [Oxoid TV5008D, Oxoid Ltd., U.K.] into the mesial canal of each premolar with a sterile tuberculin syringe inserted 1 mm short of the estimated root canal length.

The fluid was then agitated with a sterile stainless steel K-file #20 [Colorinox. Dentsply Maillefer, Ballaigues, Switzerland] (7). Any excess of sample fluid in the chamber was removed so that only the root canals remained filled. The sample fluid was then soaked from the canals with a sterile paper point [Protaper F2, Denstply Maillefer, Ballaigues, Switzerland] placed 1 mm short of the estimated root canal length and transfered to the sample fluid vial. This one constituted the first sample [S1] for groups 1, 2, 3 and for the positive control group. All samples were immediately forwarded to a certified laboratory [Laboratorio Veterinario Garfia S.L., Córdoba, Spain] for the microbiological analysis.

Then, the root canals were washed with 10 ml of sterile saline to eliminate the sample fluid. The working length [WL] was established 1 mm short of the radiographic apex, and the canals of the 3 experimental groups were disinfected with 40 ml of 5.25% sodium hypochlorite [NaOCI] using the apical negative pressure irrigation system Endovac [Endovac; Discus Dental, Culver City, CA, USA]. Taking into account that they were being treated immature teeth with open apices, the EndoVac protocol was modified to avoid the extrusion of the sodium hypochlorite to the apical tissues, as described by Cohenca *et al.* (7), briefly: first was fit the apical size of the canal and then, 40 ml 5.25% sodium hypochlorite [NaOCl] was delivered using the open-ended macrocannula in an up and down movement from a point just below the coronal orifice of the canal to the WL. To ensure that the canal persisted totally filled with the irrigant solution and no air was drawn into the canal space, the macrocannula was removed from the canal being the pulp chamber plenty of irrigant. The canals were left filled with NaOCl for 60 seconds and then were irrigated with 10 ml sterile saline solution to eliminate the rests of sodium hypochlorite and dried with sterile paper points (7).

- Group 1: QMix solution

Each canal was then flushed with 1 ml of QMix solution [Dentsply/Tulsa Dental, Johnson City, TN, USA] (8) using a sterile tuberculin syringe. The canals were left filled with the solution for 90 seconds [according to the mannufacturer recomendation] and then irrigated again with sterile saline and dried with sterile paper points.

Approximately 1 ml of sample fluid Schaedler Anaerobe Broth was delivered into the canals using a sterile tuberculin syringe inserted at the WL. Any excess of sample fluid in the chamber was removed so that only the root canals remained filled. The fluid was then agitated with a size #20 sterile stainless steel file. The sample fluid was then soaked from the canals with a sterile absorbent paper point placed in the mesial root at the WL and transfered to the sample fluid vial. This constituted the second sample [S2] for group 1. All samples were immediately forwarded to the laboratory.

All canals received a final irrigation with saline solution to eliminate the sample fluid and were dried with sterile paper points. A sterile cotton pellet was placed in the pulp chamber and the coronal access was double sealed with IRM [Caulk Co., Division Dentsply International Inc., Milford, DE, USA] and Vitrebond glass ionomer cement [3M,/ESPE, St. Paul, MN, USA].

-Group 2: QMix solution + 2% chlorhexidine gel dressing 

The teeth from Group 2 were disinfected in two sessions. The canals were irrigated with 1 ml of QMix solution with a sterile tuberculin syringe. The solution was left in the canals for 90 seconds. Then the QMix solution was eliminated with 10ml of saline solution with a sterile tuberculin syringe and the root canals were filled with the sample fluid Schaedler Anaerobe Broth with an insulin syringe. The fluid was agitated with a sterile stainless steel #20 K-file. A sterile paper point was placed in the mesial canals at working length to impregnate with the sample fluid and put it in the sample bottle. This constituted the second sample [S2] for Group 2.

Then, canals received a final irrigation with 10 ml of saline solution and were dried with sterile paper points. Immediately, 2% chlorhexidine gel [Consepsis, Ultradent Products Inc., South Jordan, UT, USA] was placed as intracanal dressing using a #4 lentulo paste carrier [Denstply Maillefer,Ballaigues, Switzerland]. All excess of chlorhexidine gel was removed and a sterile cotton pellet was placed into the pulp chamber. The coronal access was temporary restored with a doubled seal of Cavit and Vitrebond. The intracanal dressing was left for 15 days.

In a second session, all teeth from this group were isolated with a rubber dam in aseptic conditions, as described above. The coronal seal was removed with sterile high speed diamond round bur under copious water cooling followed by flushing of the pulp chamber with 20 ml saline solution to eliminate the chlorhexidine intracanal dressing. Each canal was then flushed with 2.5 ml of the combination of 3% Tween 80 [P4780 Sigma-Aldrich Quimica SA, Madrid, Spain] and 0.3% L-α-lecithin solution to neutralize the chlorhexidine residual effect (9).

The root canals were then irrigated again with saline to eliminate theTween 80 solution. Then 1 ml of sample fluid Schaedler Anaerobe Broth was delivered into the mesial canals using a sterile insulin syringe. The fluid was then agitated with a size #20 sterile stainless steel file. The sample fluid was then soaked from the canals with a sterile absorbent paper point placed in the mesial root at the WL and transfered to the sample fluid vial. This constituted the third sample [S3] for Group 2. All canals received a final irrigation and were sealed as previously explained in group 1.

- Group 3: Triantibiotic paste

The teeth from Group 3 were also disinfected in two sessions. In the first treatment session, after sodium hypochlorite irrigation, canals were flushed with 2.5 ml 5% sodium thiosulfate [Sigma-Aldrich Quimica SA, Madrid, Spain] to neutralize the sodium hypochlorite and then irrigated again with 10 ml of sterile saline. The sample fluid Schaedler Anaerobe Broth was delivered in the mesial canals and agitated with a sterile stainless steel #20 K-file. The fluid was then soaked from the canal with a sterile paper point and placed in the sample vial. This constituted the second sample [S2] for group 3. All canals received a final irrigation with 10 ml sterile saline solution to eliminate the sample fluid.

A triantibiotic paste, prepared immediately before the treatment by mixing ciprofloxacin, metronidazole and minocycline with sterile distilled water, at a concentration of 20 mg of each antibiotic, was delivered into the root canal with a #4 lentulo paste carrier. All excess of the triantibiotic paste in the pulp chamber was removed and a sterile cotton pellet was placed. The coronal access was then restored with a double sealed of Cavit and Vitrebond. The intracanal dressing was left in the canals during 15 days.

At the second treatment session, two weeks later, teeth were isolated with a rubber dam as previously described. The coronal seal was removed with sterile high speed diamond round bur under copious water cooling followed by flushing of the pulp chamber with 20 ml saline solution to eliminate the triantibiotic intracanal dressing. After that, 1 ml sample fluid Schaedler Anaerobe Broth was delivered into the mesial canals using a sterile insulin syringe. The fluid was then agitated with a size #20 sterile stainless steel file. The sample fluid was then soaked from the canals with a sterile absorbent paper point placed in the mesial root at the WL and transfered to the sample fluid vial. This constituted the third sample [S3] for Group 3. All canals received a final irrigation and were sealed as previously explained in group 1.

- Control groups

As negative control group, five premolars diagnosed with a normal healthy pulp and no radiographic signs of apical periodontitis were accessed and sampled identically to the experimental groups. These served as controls to verify the effectiveness and asepsis of the sampling technique.

Positive control group was also constituted by five premolars that were infected and sampled at S1. Then, were left untreated and irrigated only with 40 ml of isotonic saline solution and, finally, sampled at S2 and S3.

- Microbiological processing 

Samples S1, S2 and S3 were diluted in saline solution until reaching 1/10, 1/100, 1/1000, and 1/10.000 final concentrations. Then, 1 ml of each dilution were seeded using the Westergreen technique in the culture medium, Schaedler Anaerobe Agar with sheep blood [supplemented with hemin and K1 vitamin] [Oxoid PB5034A, Oxoid Ltd.,U.K.] for detection of anaerobic growth bacteria.

The dishes were placed in a jar with an anaerobiosis generator [Anaerogen Compact, Oxoid AN0010, Oxoid Ltd., U.K.] and were incubated at 37°C for 10 days. After the incubation period, the number of colony forming units CFU was counted with a colony counter [IUL Colony Counter, IUL S.A., Barcelona, Spain].

Throughout the experimental phase, all the dogs were daily monitored for signs of pain associated with the dental procedures.

- Statistical analysis

Differences were assessed between three time points [S1-S2, S2-S3, and S1-S3]. A log transformation of each CFU count was performed to normalize the data before statistical evaluation because of the high range of bacterial counts.

Data were analyzed statistically by the U-Mann-Whitney test, and a significant level of 5% was set for all the analysis.

## Results

Four teeth, two in group 2 and two in group 3, were lost due to the initial infection procedure, reducing the sample size to 8 teeth in both groups. No bacterial growth was observed from samples taken from the teeth serving as negative controls. On the contrary, bacteria were present in 100% of S1, S2 and S3 samples taken in positive controls teeth.

In the three experimental groups, all S1 samples showed bacterial growth ( Table 1). Colony-forming units [CFU] counts ranged from 27 to 270,000 [median 2,880] in group 1, from 1,188 to 3,456,000 [median 22,860] in group 2, and from 9 to 3,456,000 [median 25,200] in group 3. There was no statistically significant difference between the groups [*p* > 0.05].

**Table 1:**
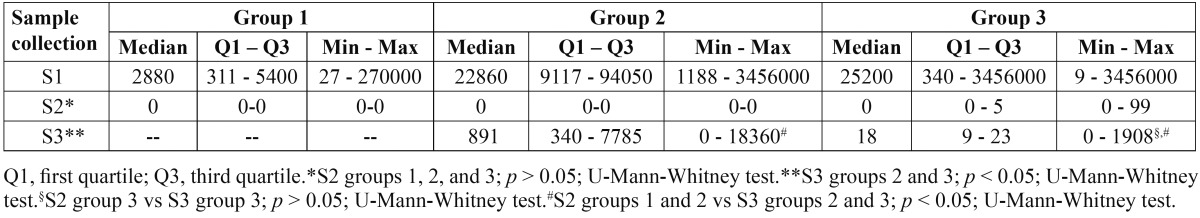
Colony-forming units counts in the first (S1), second (S2) and third (S3) samples collection of groups 1 (QMix solution; n = 10), 2 (QMix solution + 2% chlorhexidine gel dressing, for 15 days; n = 8), and 3 (triantibiotic paste, for 15 days; n = 8).

After the first session of the disinfection protocol, there was significant [*p* < 0.05] bacterial reduction from S1 to S2 in the three experimental groups. Microorganisms were absent in 100% of S2 samples in groups 1 and 2, and in 75% of the teeth of group 3 [range 0 to 99 cfu, median 0], but there was no statistically significant difference between the groups [*p* > 0.05].

Finally, the S3 samples, taken after dressing in groups 2 and 3, showed an increased bacterial count. In both groups, 87.5% of teeth showed bacterial growth. In group 2, CFU counts [range = 0 - 18,360; median 891] were significantly higher than in group 3 [range = 0 - 1,908; median 18] [p = 0.03], indicating that triantibiotic paste dressing was more effective in bacterial reduction than chlorhexidine dressing.

There were significant differences between S2 samples of group 1 and 2 and S3 samples of group 2 and 3 [*p* < 0.05], indicating that 40 ml 5.25% sodium hypochlorite, using Endovac system, plus a final irrigation with 1 ml QMix solution, provides a higher bacterial reduction that two treatment sessions with intracanal dressing of 2% chlorhexidine gel or triantibiotic paste.

## Discussion

In this study, three different disinfection protocols have been assessed in vivo in immature dog teeth with pulp necrosis and apical periodontitis. The results showed that root canal irrigation with 5.25% sodium hypochlorite alone, using Endovac system in one session, provides the same level of disinfection than when using additionally a QMix solution, with or without chorhexidine gel dressing, or a triantibiotic paste dressing.

In order to achieve the success of the endodontic therapy, it is required to eliminate bacteria from all infected canals, especially in teeth with pulp necrosis and periapical lesions (2). Moreover, periapical repair and tissue healing only can occur after reducing sufficiently the microbial load in root canals (10). Nowadays, most of disinfection protocols include conventional irrigation with 4-6% sodium hypochlorite, with or without dressing with a triantibiotic paste (11), and apical negative pressure irrigation (12). It has been demonstrated that apical negative pressure irrigation eliminates more microorganisms from the root canals than the traditional apical positive irrigation (7,12).

Different disinfection techniques have been proposed for immature teeth with apical periodontitis, however it has not been definitively established which one provides the best results (13). Most of the irrigation protocols include sodium hypochlorite solution due to its effectiveness eliminating organic tissue and its broad antibacterial spectrum. Sodium hypochlorite neutralizes bacteria, fungus, spores and viruses [HIV, Hepatitis A and B] (14). The apical negative pressure irrigation system Endovac, appears to be safe to use in immature teeth because it prevents the irrigation solution to extrude to the periapical tissues (15). Some authors recommend conducting a final irrigation with QMix solution to eliminate the smear layer from the dentin walls and to provide additional disinfection because QMix contains EDTA and chlorhexidine (8). The QMix solution provides superior efficacy eliminating inorganic wastes and erodes less dentin than 17% EDTA solution (8). In this study, the disinfection with 40 ml 5.25% sodium hypochlorite, using the apical negative pressure system Endovac, plus a final irrigation with QMix solution in a unique session, eliminates 100% of microorganisms in immature dog premolars with apical periodontitis. Nevertheless, root canal irrigation with 5.25% sodium hypochlorite, using EndoVac system, alone, achieves similar results.

It has been claimed that the use of medical dressings in the endodontic procedure might reduce the bacterial flora below the limits reached only with the endo treatment, mainly because they access to areas of the root canal system where irrigation cannot (16). Thus, several investigators (16,17) have found that the percentage of success in infected root canals decreases when there is no use of intracanal dressing. Therefore, chlorhexidine has been used not only as irrigant but also as intracanal dressing (18), because it is bactericide, has sustantivity and low toxicity, and adapts to the irregularities of the root canal (19), but it cannot solve the organic tissue (20-22). After exposing the root canal to chorhexidine for at least one week, the medicament has a residual antimicrobial activity of 72 to 168 hours (18), but the residual gel makes the sealing of the root canal more difficult during obturation (23). Alternatively, triantibiotic paste [ciprofloxacin, metronidazole and minocycline] dressing has been used to reduce the number of bacteria in the root canal (24,25). But the use of antibiotic pastes could result in development of strain resistance (26,27), allergic reactions (28) and decolorations (29,30).

On the contrary, in the present study the disinfection with sodium hypochlorite, using Endovac system, plus a final irrigation with QMix solution, provides a significantly higher bacterial reduction that two treatment sessions with intracanal dressing of 2% chlorhexidine gel or triantibiotic paste. This result suggests that bacteria reduction is higher when treatment is carried out in only one session, probably because, as it can be seen table 1, there is a slight bacteria growth when the intracanal dressing is left in the canals for 15 days. This could be the result of an apical bacterial micro leakage and as an effect of the flushing of the dressing through the open apices. However, triantibiotic paste dressing was significantly more effective in bacterial reduction than chlorhexidine dressing. Thereafter, if an intracanal dressing must be used, it is more effective to leave the triantibiotic paste than 2% chlorhexidine gel.

The results of the present study demonstrated that, root canal irrigation with 5.25% sodium hypochlorite alone, using Endovac system, in only one session, provides the same level of disinfection than when using additionally a QMix solution, with or without chorhexidine gel dressing, or a triantibiotic paste dressing. It can be conclude that neither QMix solution or intracanal dressing with chlorhexidine gel or triantibiotic paste, improve root canal disinfection of immature dog teeth with apical periodontitis.
